# Wheat COBRA-like Gene *TaCOBL6A2* Confers Heat Tolerance in Plants

**DOI:** 10.3390/ijms26094101

**Published:** 2025-04-25

**Authors:** Qingyan Deng, Jiangtao Luo, Jianmin Zheng, Peixun Liu, Dejun Wang, Zongjun Pu

**Affiliations:** Key Laboratory of Wheat Biology and Genetic Improvement on Southwestern China of MARA, Environment-friendly Crop Germplasm Innovation and Genetic Improvement Key Laboratory of Sichuan Province, Crop Research Institute, Sichuan Academy of Agricultural Sciences, Chengdu 610066, China; dengqy2020@163.com (Q.D.); jtluohao@163.com (J.L.); jianminxingzheng@yeah.net (J.Z.); littlefarmer@163.com (P.L.); wangdj2025@163.com (D.W.)

**Keywords:** COBRA-like, *TaCOBL6A2*, heat tolerance, haplotype, wheat

## Abstract

Wheat, a cold-tolerant crop, suffers substantial yield and quality losses under heat stress, yet the genetic mechanisms underlying thermotolerance remain understudied. We characterized *TaCOBL6A2*, a novel COBRA-like gene on wheat chromosome 6A encoding a glycosylphosphatidylinositol (GPI)-anchored protein with a conserved COBRA domain, and performed subcellular localization, tissue-specific expression, and stress response analyses to investigate its function. Functional validation was conducted based on *TaCOBL6A2* overexpression in *Arabidopsis* and transcriptomic profiling. Additionally, a haplotype analysis of wheat varieties was performed to associate genotypes with heat stress phenotypes. The results show that TaCOBL6A2 is localized to the plasma membrane, the cell wall, and the nucleus, with the highest expression in early-stage grains. Its transcription was strongly induced by heat stress, exceeding that in response to cold, salt, or drought. Its overexpression in *Arabidopsis* enhanced thermotolerance and activated heat shock proteins (HSPs) and oxygen homeostasis pathways. The elite haplotype, Hap1, was associated with improved seedling growth and elevated antioxidant enzyme activity under heat stress. Our findings reveal that *TaCOBL6A2* is a key regulator of wheat heat tolerance and could be used as a molecular target for breeding climate-resilient cultivars.

## 1. Introduction

Wheat (*Triticum aestivum* L.), a globally crucial staple food, phylogenetically originates from the Fertile Crescent in Western Asia [1]. Characteristically, it is a cold-tolerant crop and exhibits a relatively low adaptability to high-temperature conditions [2,3,4]. Therefore, the increase in global temperature has become a major threat to global wheat production. It is estimated that a 1 °C rise in global temperature could lead to a 6% reduction in the harvest index of a crop [5]. High temperatures affect crop growth and yields through direct and indirect effects, with diverse consequences, including shorter growth and development periods, cooling responses, changes in grain and seed characteristics, early or restricted seed setting, and accelerated senescence of photosynthetic organs [6]. Thus, it is crucial to investigate the stress response mechanisms in wheat to enhance its heat tolerance, thereby facilitating increased crop productivity.

The heat tolerance of plants is a complex biological characteristic. Genes implicated in its regulation, along with their corresponding physiological and biochemical processes, are highly intricate and diverse. In recent years, a large number of heat stress defense genes have been documented, including cellular stress proteins such as heat shock proteins (HSPs) [7,8,9] and transcription factors such as heat shock transcription factors (HSFs) [10,11,12], dehydration response element binding factor (DREB) [13], and multiprotein bridging factor 1 (MBF1) [14,15], along with a multitude of other protein responses to heat stress [16,17,18]. In addition, emerging evidence indicates that plant cell wall remodeling plays a crucial role in the response to heat stress through the activation of cell wall-related genes and the alteration in cell wall compositions [19]. For example, the expansin gene *AsEXP1* has been reported to be associated with heat tolerance [20], and the overexpression of *PpEXP1* in tobacco enhanced the plant’s tolerance to heat stress [21]. In *Arabidopsis*, *hot2* was found to encode a chitinase-like protein required for ordered cellulose deposition and was shown to be essential to tolerance to heat, salt, and drought stress [22]. In addition, the pectin methylesterase gene *AtPME34* modulates guard cell wall flexibility to mediate the heat response in *Arabidopsis* [23].

Generally, heat stress-responsive genes play a crucial role in enhancing plant thermal tolerance through overexpression mechanisms, potentially conferring greater adaptability and survival advantages under conditions of elevated temperature. In maize, two key regulatory genes, *ZmWRKY106* and *ZmCDPK7*, were significantly expressed by high-temperature stress, conferring enhanced heat tolerance to both transgenic *Arabidopsis* and maize plants, respectively [18,24]. Similarly, in wheat, the overexpression of *TaHsfA2e-5D*, a heat shock transcription factor, and *TaPEPKR2*, a calcium-dependent protein kinase, conferred significant heat tolerance to both *Arabidopsis* and wheat plants [11,17]. Furthermore, the stress granule-associated gene *TaMBF1c*, which encodes a transcriptional coactivator, was shown to contribute substantially to heat tolerance in wheat, with its overexpression lines exhibiting markedly improved resistance to heat stress compared with wild-type plants [15]. These findings collectively highlight the conserved molecular mechanisms underlying heat stress responses across different plant species and provide valuable genetic resources for developing heat-tolerant crop varieties with genetic engineering methods.

However, the genetic basis underlying plant thermotolerance remains largely unexplored, with numerous heat-responsive genes yet to be identified and functionally characterized. Among the potential candidates, plant-specific COBRA-like (*COBL*) genes, which encode glycosylphosphatidylinositol-anchored proteins (GPI-APs) [25], have emerged as crucial regulators in diverse biological processes. Structurally, COBL proteins contain an N-terminal secretion signal sequence, N-glycosylation sites, a conserved Cys-rich (CCVS) motif, and an ω-attachment site for GPI modification along with a hydrophobic C terminus [26]. Functionally, COBL proteins regulate anisotropic cell expansion by modulating cellulose biosynthesis. Biochemical studies demonstrate their direct binding to cellulose synthase (CESA) complexes, which influences microfibril crystallinity and deposition patterns in both primary and secondary cell walls [27,28,29]. They are extensively involved in plant growth and development, particularly in cell wall formation [30], root and grain development [31,32], as well as gametophyte development [33]. Beyond developmental roles, emerging evidence suggests that *COBL* genes participate in the response to abiotic stress including heat, cold, salinity, and drought stress. Comparative genomic analyses have revealed that COBL family members in *Arabidopsis*, *Zea mays*, and *Populus* exhibit differential expression patterns and improved stress adaption under various stress conditions [34,35,36]. In *Arabidopsis*, the overexpression of *COBL9* could promote root hair elongation and salinity tolerance [31]. In wheat, five *TaCOBLs* were significantly down-regulated under drought treatment [36], whereas in rice, *DROT1* (a COBL homolog) was shown to enhance drought resistance through cell wall modification by increasing cellulose content and maintaining cellulose crystallinity [37]. Despite these advances, the specific regulatory mechanisms of COBL family genes in response to high-temperature stress remain poorly understood, representing a critical knowledge gap in plant stress biology.

In this study, we identified a novel wheat *COBL* gene on chromosome 6A and designated it as *TaCOBL6A2*. As it was significantly up-regulated under heat stress treatment, we investigated its expression patterns, subcellular localization, transcriptomic alteration, elite haplotype, antioxidant enzyme activity, and stress tolerance in transgenic *Arabidopsis* and wheat germplasms to elucidate its biological function in response to heat stress. The findings provide evidence that *TaCOBL6A2* plays a significant role in plant responses to heat. Additionally, the development of functional markers corresponding to the *TaCOBL6A2* elite haplotype is of great significance to the breeding of heat-tolerant wheat in the future.

## 2. Results

### 2.1. Genomic Identification and Evolutionary Analysis of TaCOBL6A2

**Figure 1:**
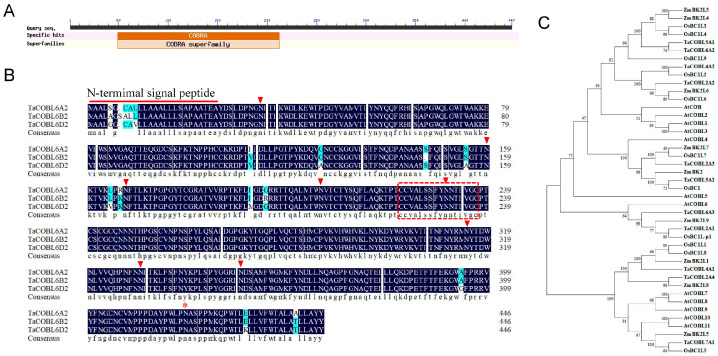
Conserved domain architecture and phylogenetic relationships of TaCOBL6A2. (**A**) The conserved domain of TaCOBL6A2. (**B**) Multiple sequence alignment of TaCOBL6A2 and its homologs. Triangle and asterisk represent N-glycosylation site and ω-site, respectively. The red dashed boxed sequences denote the Cys-rich CCVS motif. (**C**) Phylogenetic tree of TaCOBL6A2 and COBL family members from *A. thaliana, Oryza sativa*, and *Zea mays*.

In our prior genomic investigation, we systematically identified the plant-specific COBL gene family in wheat. Three homoeologous genes, namely *TaCOBL6A2*, *TaCOBL6B2*, and *TaCOBL6D2*, were located on chromosome 6. These genes encode a protein that contains a conserved COBRA domain (Figure 1A), an N-terminal signal peptide, potential N-glycosylation sites, a conserved Cys-rich CCVS domain, and a hydrophobic C terminus that contains the ω-attachment site for GPI modification (Figure 1B). Sequence alignment based on the publicly available Chinese Spring reference proteome (IWGSC RefSeq v2.1) revealed exceptional conservation among homologs, with TaCOBL6A2 displaying only sporadic amino acid substitutions compared with its paralogs (Figure 1B).

To elucidate the evolutionary relationships, we conducted phylogenetic reconstruction by using a neighbor-joining algorithm on multi-aligned protein sequences encompassing 12 *Arabidopsis* (AtCOBLs), 11 rice (OsCOBLs), and 9 maize (ZmCOBLs) orthologs (Figure 1C). According to the phylogenetic tree, all COBLs were classified into two groups. The *TaCOBL6A2*-encoding COBL protein showed the best similarity to rice BC1L3/BC1L4 and maize BK2L3/BK2L4 and was clustered in a monocot-specific clade with three ZmCOBLs and five OsCOBLs. Conversely, four Arabidopsis COBLs (AtCOB and AtCOBL1-3) formed a dicot-specific lineage. These results indicated that an ancient COBL gene family existed before the separation of monocotyledons and dicotyledons, after which its function may have differentiated.

### 2.2. Subcellular Localization of TaCOBL6A2 Reveals Tripartite Targeting of the Plasma Membrane, Cell Wall, and Nucleus

**Figure 2:**
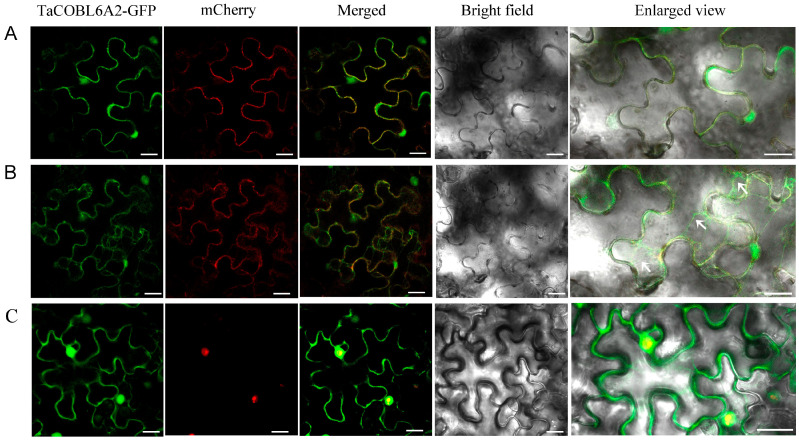
Subcellular localization of TaCOBL6A2 in tobacco epidermal cells. (**A**) Plasma membrane localization of TaCOBL6A2 in tobacco epidermal cells. (**B**) Mannitol-induced plasmolysis to examine the cell wall localization of TaCOBL6A2 in tobacco epidermal cells. (**C**) Nuclear localization of TaCOBL6A2 in tobacco epidermal cells. The OsPIP1;3-mCherry and OsMADS57-mCherry were used as a plasma membrane and nuclear localization markers, respectively. Panels show GFP, mCherry, and their merged images, along with the bright field. The far right panel displays an enlarged view of the cells merged with GFP, mCherry, and bright field. The arrow indicates the plasma membrane after plasmolysis. Bars = 25 μm.

We performed transient expression assays in tobacco leaf epidermal cells to elucidate the subcellular localization of the TaCOBL6A2 protein. The TaCOBL6A2–GFP fusion construct was co-expressed with OsPIP1;3-mCherry, a well-characterized plasma membrane marker [38]. In the transformed leaf epidermal cells, TaCOBL6A2–GFP co-localized with OsPIP1;3-mCherry on the plasma membrane (Figure 2A). We further induced plasmolysis by using 1 M mannitol treatment to further discriminate between plasma membrane and cell wall localization. The plasmolysis experiment demonstrated that TaCOBL6A2–GFP fluorescence was retained in both the plasma membrane and cell wall compartments (Figure 2B). Interestingly, we also observed GFP fluorescence in the nuclear compartment. To validate this nuclear localization, we co-expressed TaCOBL6A2–GFP with the nuclear marker OsMADS57-mCherry [39], which showed clear co-localization in the nucleus (Figure 2C). Collectively, these findings demonstrate that TaCOBL6A2 exhibits a unique tripartite localization pattern, targeting the plasma membrane, the cell wall, and the nucleus.

### 2.3. Expression Profiling and Stress Response Analysis of TaCOBL6A2

The transcriptomic analysis of Chinese Spring wheat revealed that *TaCOBL6A2* and its homologous genes (*TaCOBL6B2* and *TaCOBL6D2*) exhibit constitutive expression across multiple tissues, including roots, stems, leaves, spikes, and grains, in various developmental stages. Notably, their expression profiles showed significant up-regulation during the early grain formation stage (Figure S1). Moreover, the homologous genes located on the ABD subgenomes displayed similar expression patterns. We selected *TaCOBL6A2* (A subgenome) as the representative gene for subsequent functional characterization based on the high sequence conservation (Figure 1B) and synchronized expression profiles of these homologous genes.

**Figure 3:**
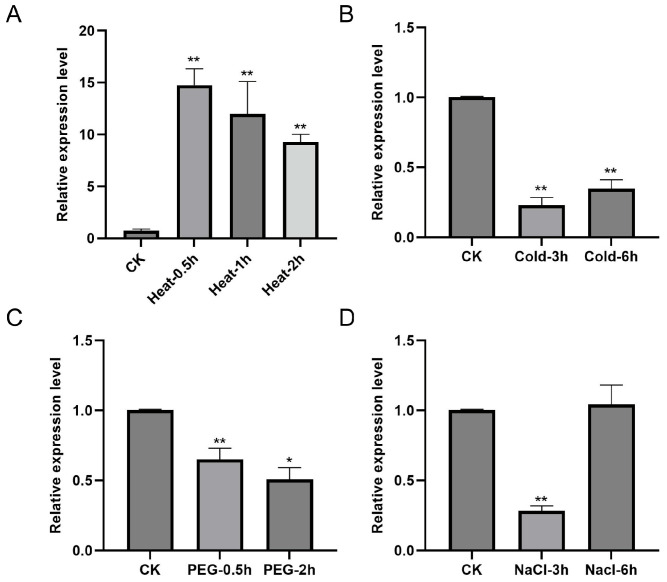
Expression of *TaCOBL6A2* under (**A**) heat, (**B**) cold, (**C**) drought, and (**D**) salt stress. Vertical bars represent the SE of the mean (*n* = 3). *, ** represent statistically significant differences at *p* < 0.05 and *p* < 0.01.

We conducted time-course expression analyses under four major stress conditions to investigate the potential role of *TaCOBL6A2* in abiotic stress responses: heat (42 °C), cold (4 °C), drought (simulated with 30% PEG-6000 treatment), and salt (250 mM NaCl) stress. Quantitative RT-PCR analysis revealed that *TaCOBL6A2* expression was rapidly induced by heat stress, showing a significant 14-fold increase 0.5 h post-treatment, followed by gradual 11-fold and 9-fold decreases at 1 h and 2 h, respectively (Figure 3A). In contrast, cold stress and PEG-induced drought stress resulted in substantial downregulation of *TaCOBL6A2* expression throughout the treatment period (Figure 3B,C). Under salt stress conditions, *TaCOBL6A2* expression exhibited transient suppression at 3 h (by 0.7 times of the control) followed by recovery to basal levels at 6 h (Figure 3D). These differential expression patterns suggest that *TaCOBL6A2* may play distinct roles in plant responses to various abiotic stress conditions, with particularly significant involvement in heat stress adaptation.

### 2.4. Overexpression of TaCOBL6A2 Enhances Thermotolerance in Arabidopsis

**Figure 4:**
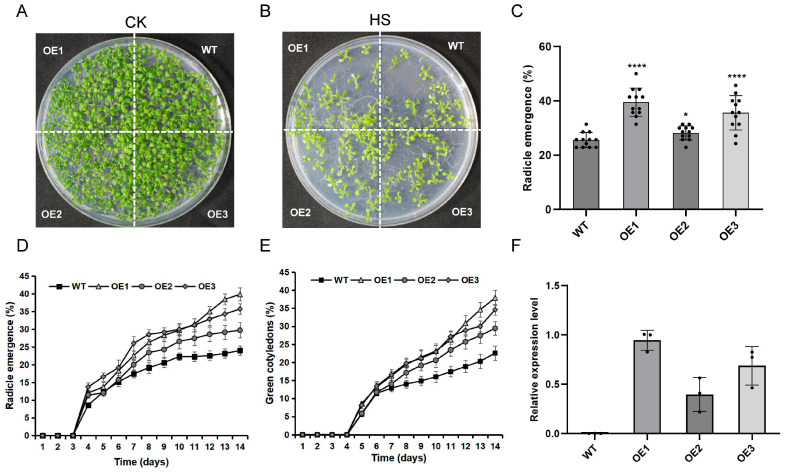
Characterization of wildtype (WT) and *TaCOBL6A2* transformants. (**A**,**B**) Morphology of 14-day-old seedlings of WT and three overexpression lines (OE) under control condition and heat stress (HS) treatment. (**C**) Germination levels (radicle emergence parameter) of WT and OE lines after HS treatment. (**D**,**E**) Radicle emergence and green cotyledons emergence during 14 days post-heat shock period. (**F**) The relative expression of TaCOBL6A2 in WT and three OE lines. Data are presented as mean ± SE (*n* = 12). *, *p* < 0.05; ****, *p* < 0.0001.

To investigate the functional role of *TaCOBL6A2* in thermotolerance, we conducted heat stress assays by using three independent transgenic *Arabidopsis* lines overexpressing *TaCOBL6A2* under the control of the CaMV 35S promoter. Surface-sterilized seeds were subjected to a heat shock treatment (50 °C for 1 h) in a water bath prior to germination under controlled conditions (a 22 °C/18 °C day/night temperature regime). Following 14 days of germination, the heat-stressed (HS) seedlings exhibited significantly reduced survival rates compared with the non-stressed control (CK) (Figure 4A,B). All three overexpression (OE) lines demonstrated enhanced thermotolerance relative to the wild-type (WT), as evidenced by higher germination levels (Figure 4C). Moreover, the daily monitoring of radicle emergence and green cotyledon development revealed similar trends, with OE lines displaying accelerated recovery dynamics (Figure 4D,E). Importantly, we observed a strong positive correlation between *TaCOBL6A2* transcript levels and seed germination rates across the transgenic lines (Figure 4C,F), suggesting a dose-dependent effect of *TaCOBL6A2* expression on thermotolerance. These results demonstrate that the overexpression of *TaCOBL6A2* significantly enhanced the thermotolerance of *Arabidopsis*.

### 2.5. Comparative Transcriptomic Analysis of TaCOBL6A2 Overexpression Lines

To further elucidate the transcriptomic alterations in *TaCOBL6A2* OE lines under heat stress, we collected survival seedlings of transgenic *Arabidopsis* and WT plants after heat-treated seed germination and twelve samples (three biological replicates per group) were subjected to RNA-seq analysis (Supplementary File S1).

The differential expression analysis revealed 1825 significantly up-regulated and 613 down-regulated genes in WT plants following heat stress treatment (Figure S2A). In contrast, the *TaCOBL6A2* OE lines exhibited a substantial reduction in the number of up-regulated genes (707) and a slight increase in that of down-regulated genes (762) (Figure S2B), indicating that the wheat *TaCOBL6A2* gene altered the transcriptomic response of *Arabidopsis* to heat stress. GO enrichment analysis demonstrated that heat stress induced enrichment of biological processes related to detoxification (e.g., drug catabolic process and cellular oxidant detoxification) and stress responses (e.g., responses to hypoxia and toxic substances) in both the WT and *TaCOBL6A2* OE lines (Figure S2C,D). KEGG pathway analysis further identified plant hormone signal transduction and phenylpropanoid biosynthesis as the most significantly altered pathways in both groups (Figure S2E,F).

**Figure 5:**
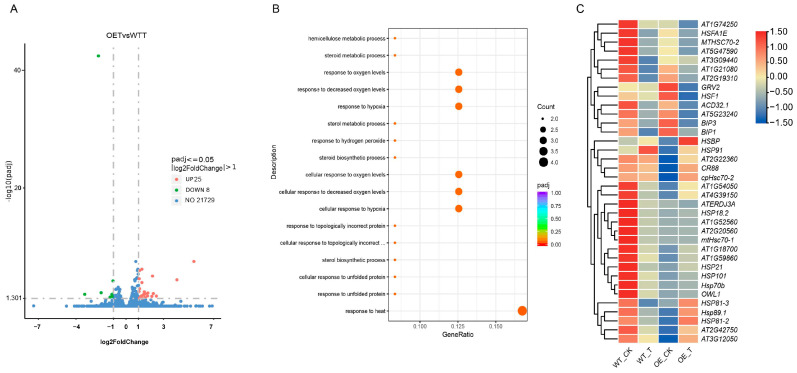
DEG analysis of wild-type and *TaCOBL6A2* overexpression (OE) lines under heat stress treatment. (**A**) The volcano indicating the DEGs in wild-type and *TaCOBL6A2* OE lines under heat stress treatment. (**B**) Scatter plots of GO enrichment statistics between wild-type and *TaCOBL6A2* OE lines under heat stress treatment. (**C**) The heat map of heat shock genes and its regulation genes in wild-type and *TaCOBL6A2* OE lines. The FPKM values of genes from transcriptome databases were used to construct the heatmap. The colors represent relative expression levels normalized by row scale. These genes were selected according to *p* < 0.05 in OE_T vs. OE_CK or WT_T vs. WT_CK.

A comparative analysis of the WT and *TaCOBL6A2* OE lines under heat stress conditions identified only 33 differentially expressed genes (DEGs) (Table S1), with 25 being up-regulated and 8 being down-regulated (Figure 5A). Notably, the GO enrichment of these DEGs highlighted significant involvement in the response to heat and oxygen levels (Figure 5B). Key heat-responsive genes included *HSP70*, *MBF1c*, *HSFA2*, and *AT1G5986* (encoding an HSP20-like chaperone), suggesting that *TaCOBL6A2* modulates adaptability to heat stress and oxygen fluctuations. A further analysis of the heat shock protein (HSP)-related DEGs revealed distinct expression patterns between the WT and OE lines. Most known HSP genes (e.g., *CR88*, *HSP81-2*, *HSP81-3*, *cpHsc70-2*, and *HSP89.1*) were down-regulated in the WT but up-regulated in the *TaCOBL6A2* OE lines after heat treatment (Figure 5C, Table S2). Strikingly, no significant DEGs showed up-regulation in the WT and down-regulation in the OE lines, underscoring the specific role of *TaCOBL6A2* in enhancing HSP expression.

**Figure 6:**
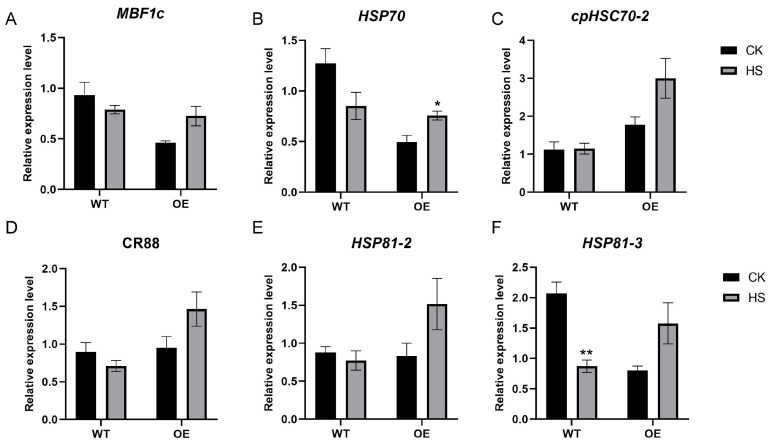
RT-qPCR analysis of heat shock genes and its regulation genes in wild-type (WT) and TaCOBL6A2 OE lines under heat stress (HS). (**A**) *MBF1c*, (**B**) *HSP70*, (**C**) *cpHSC70-2*, (**D**) *CR88*, (**E**) *HSP81-2*, (**F**) *HSP81-3* were examined under control and heat stress conditions. Expression levels of target genes were calculated using the 2^−ΔΔCt^ method, with non-HS-treated seedlings as the control. Data represent mean values ± SE from three biological replicates. *, ** indicate statistically significant differences at *p* < 0.05 and *p* < 0.01, respectively.

We performed an RT-qPCR analysis on several key HSP genes to validate the RNA-seq results. Under heat stress conditions, the transcripts of all detected HSP genes in WT plants were reduced, except for *cpHSC70-2* (Figure 6 A–F). Notably, *HSP81-3* exhibited significant down-regulation (*p* < 0.01) (Figure 6F). In the OE lines, all examined HSP transcripts were induced during heat stress, with *HSP70* demonstrating particularly significant up-regulation (*p* < 0.05) (Figure 6B). Overall, the RT-qPCR results show strong concordance with our RNA-seq data.

Collectively, these results demonstrate that *TaCOBL6A2* overexpression enhances thermotolerance in *Arabidopsis* by reprogramming the expression of heat shock genes (e.g., HSPs) and their regulatory factors.

### 2.6. Natural Variation and Elite Haplotypes of TaCOBL6A2 Enhance Thermotolerance in Wheat

To identify natural allelic variations and the elite haplotypes of *TaCOBL6A2* in wheat, we performed a haplotype analysis on 278 sequenced wheat accessions from public databases. The analysis of sequence variations in the coding region, introns, and untranslated regions (UTRs) revealed five polymorphic sites (located in the 5′UTR, exon 3, exon 6, and 3′UTR), defining three distinct haplotypes (Hap1, Hap2, and Hap3) (Figure S3A), which were further validated by using two KASP markers (Figure S3B,C) in 175 wheat varieties/lines (Table S3); Hap1, Hap2, and Hap3 were detected in 116, 44, and 15 accessions, respectively.

**Figure 7:**
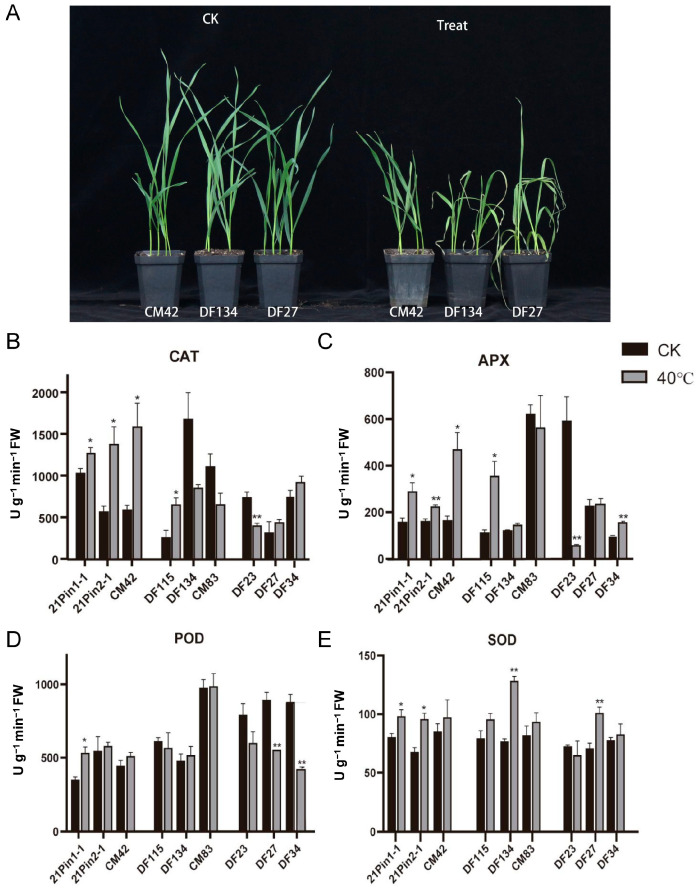
Phenotypic variation and antioxidant enzyme activities of *TaCOBL6A2* haplotypes under heat stress. (**A**) Phenotypic responses of three haplotypes under heat stress. Representative varieties (lines) for each haplotype are shown: CM42 (Hap 1), DF134 (Hap 2), and DF27 (Hap 3). Each haplotype included three varieties (lines), with three biological replicates per variety. (**B**–**E**) Activities of antioxidant enzymes in the three haplotypes: catalase (CAT, (**B**)), ascorbate peroxidase (APX, (**C**)), peroxidase (POD, (**D**)), and superoxide dismutase (SOD, (**E**)). Hap 1: 21Pin1-1, 21Pin2-1, CM42; Hap 2: DF115, DF134, CM83; Hap 3: DF23, DF27, DF34. Asterisks indicate significant differences between control and treatment (* *p* < 0.05; ** *p* < 0.01).

To further evaluate the functional significance of these haplotypes, nine representative varieties/lines (three per haplotype) were subjected to heat tolerance assays. In comparison with the control group, the growth of the seedlings that had been treated with high temperature for one week was conspicuously inhibited. However, in the treatment group, Hap1 seedlings exhibited significantly less wilting than Hap2 and Hap3 seedlings (Figure 7A). The physiological analysis of antioxidant enzyme activity under heat stress revealed haplotype-specific responses: Catalase (CAT) and ascorbate peroxidase (APX) activities were significantly elevated in the Hap1 lines (*p* < 0.05) (Figure 7B,C). Peroxidase (POD) activity increased exclusively in the Hap1 lines, with a marked enhancement in 21Pin 1-1 (Figure 7D). Superoxide dismutase (SOD) activity increased across all haplotypes, but the highest levels were observed in two Hap1 lines (Figure 7E). Furthermore, we analyzed *TaCOBL6A2* expression across all haplotype varieties/lines (Hap1–Hap3). The results demonstrated significant up-regulation in all varieties/lines under heat stress, with the exception of DF23 (Figure S4). This observation suggests that while transcriptional induction contributes to the thermotolerance observed in Hap1, other factors, such as potential structural variations in TaCOBL6A2, may also play a role. Further investigation will be required to elucidate these additional mechanisms. These findings collectively demonstrate that Hap1 represents the elite haplotype of TaCOBL6A2, conferring enhanced thermotolerance through improved antioxidant capacity.

## 3. Discussion

The plant cell wall, a complex polysaccharide network, provides structural stability and serves as a physical barrier against biotic and abiotic stress. However, the functions and mechanisms of specific cell wall components involved in thermotolerance remain poorly understood. Previous studies have highlighted cell wall remodeling as a key adaptive response to heat stress, essential to maintaining cellular integrity and growth [19]. Nevertheless, little is known about the genes involved in plant cell wall formation and their role in the heat stress response. Among these, the COBRA-like family genes, known for their role in cellulose microfibril organization and cell wall formation [28,29,30], have not been extensively studied in the context of heat stress. Here, we provided evidence that *TaCOBL6A2*, a member of the COBRA-like family, responds to heat stress and plays a critical role in enhancing heat tolerance in both *Arabidopsis* and wheat.

The COBRA-like family is characterized by three conserved domains: a N-terminal signal peptide for secretion, a Cys-rich CCVS domain, and a hydrophobic C-terminal domain for GPI anchoring [26]. TaCOBL6A2 possesses all these domains and exhibits high sequence similarity and similar expression patterns to its homologs. In addition, *TaCOBL6A2* and its homologs are highly expressed during early grain formation, suggesting a conserved role in grain development. The cis-regulatory element analysis of *TaCOBL6A2* and its homologs revealed limited known sites directly associated with the heat stress response (Table S4). However, key motifs, such as STRE (Stress-Responsive Element), the DRE core (DREB-binding site), and the MYB-binding site have been reported to play crucial roles in stress regulation in plants [13,40,41]. The presence of these shared regulatory elements suggests that *TaCOBL6A2* and its homologs may exhibit coordinated responses to abiotic stress, despite showing some variation in expression patterns under specific stress conditions. Our expression profiling demonstrated that TaCOBL6A2 responds to multiple abiotic stress conditions, including heat, cold, drought, and salt stress, with particularly strong induction under heat stress (Figure 3). This multi-stress responsiveness aligns with previous reports indicating that certain COBL family members enhance abiotic stress adaptation by promoting cellulose deposition and cell wall remodeling [31,37]. The mechanical support provided by the cell wall may help maintain cellular integrity under osmotic stress conditions, as most abiotic stress types (including heat, drought, and salt) ultimately disrupt cellular water potential and turgor pressure homeostasis [42]. Functional characterization based on heterologous overexpression in *Arabidopsis* demonstrated that *TaCOBL6A2* significantly enhances thermotolerance, establishing its pivotal role in the heat stress response. While these findings position TaCOBL6A2 as a promising candidate for improving heat tolerance in crops, its potential roles in cold, drought, and salt stress adaptation require further mechanistic validation.

The phylogenetic analysis revealed that *TaCOBL6A2* clusters with other COBRA-like family members into two distinct groups, consistently with previous studies [27,34]. The COBRA gene family is an ancient family that emerged prior to the divergence of monocotyledon and dicotyledon phyla. To date, the functions of many members remain unknown, and uncovering the functions of all members of this family represents a formidable challenge. TaCOBL6A2 was clustered into a subclade with eight genes from rice and maize, half of which have well-defined roles in cell wall formation. For instance, ZmBk2L3 and OsBC1L4 are essential to cellulose biosynthesis during primary cell wall formation [28,43], while OsBC1L6 modulates β-glucan synthesis during endosperm cell wall formation by interacting with cellulose moieties on the plasma membrane during seed ripening [44]. Additionally, DROT1 (OsBC1L9) enhances drought resistance by modulating the cell wall structure with increased cellulose content and maintaining cellulose crystallinity [37]. TaCOBL6A2 exhibits a close phylogenetic relationship with DROT1, suggesting functional similarities. Given the overlapping regulatory networks activated by drought and heat stress [45], it is plausible that TaCOBL6A2 confers heat tolerance through mechanisms analogous to those of DROT1. However, further experimental validation is required to confirm this hypothesis and fully elucidate the molecular basis of TaCOBL6A2-mediated thermotolerance.

Generally, the overexpression of stress-responsive genes is a well-established strategy to enhance plant tolerance to environmental stress. In this study, the overexpression of *TaCOBL6A2* in *Arabidopsis* significantly improved heat tolerance, accompanied by substantial transcriptomic changes under heat stress. Notably, the number of up-regulated genes was markedly reduced in transgenic lines compared with wild-type plants, indicating a reprogramming of the heat stress response. Functional enrichment analysis showed that the DEGs were significantly associated with biological processes related to toxic substance detoxification and oxygen level regulation in both the wild-type and transgenic lines after exposure to heat stress. Simultaneously, the biological processes associated with responses to heat and oxygen levels exhibited enrichment in the DEGs of the wild-type and transgenic lines following heat stress treatment.

Previous research has proven that oxygen can produce toxic molecules known as reactive oxygen species (ROS). As oxidative stress increases in the plant, the scavenging or detoxification of excessive ROS is accomplished by an efficient antioxidative system, which includes the enzymes SOD, CAT, APX, POD, and glutathione peroxidase (GPX) [46]. Heat stress accelerates the production of ROS, and excessive ROS accumulation leads to oxidative damage, including biomolecular damage and apoptosis [47,48]. Maintaining a high antioxidant capacity for scavenging toxic ROS has been associated with enhanced tolerance of plants to heat stress. Here, wheat seedlings carrying the elite haplotype (Hap1) showed significantly enhanced heat tolerance, accompanied by elevated activity of key antioxidant enzymes, including CAT, APX, POD, and SOD (Figure 7B–E). Seedlings attempt to tolerate the stress by increasing the activity of antioxidant enzymes to maintain homeostasis between the generation and the scavenging of ROS [49].

Heat shock proteins (HSPs) and reactive oxygen species (ROS)-scavenging enzymes are two key classes of functional proteins induced by heat stress. These proteins are regulated by heat stress-responsive transcription factors and play critical roles in plant thermotolerance. Notably, the observed nuclear localization of TaCOBL6A2 (Figure 2C) suggests two potential regulatory mechanisms: (1) direct interaction with HSP gene promoters or (2) the mediation of heat stress signaling via transcription factors. The precise mechanistic relationship between TaCOBL6A2 and the regulation of HSP/ROS-related genes will be systematically investigated in subsequent studies. Numerous studies have demonstrated that the expression levels of HSPs play a pivotal role in plant survival under heat stress. We identified multiple HSPs and their regulatory factors with RNA-seq analysis, finding a significantly higher proportion of up-regulated genes in the *TaCOBL6A2* overexpression lines than in the wild-type plants under heat stress (Table S5). Specifically, in the wild-type plants, only 2 genes were up-regulated, and 34 were down-regulated, whereas in the transgenic lines, 22 genes were up-regulated, and 14 were down-regulated (Figure 5C).

Among the DEGs, several well-characterized HSPs and their regulators, including *CR88*, *HSP89.1*, *HSP70*, *MBF1C*, and *HSFA2*, were significantly up-regulated in the transgenic lines. These genes are known to be activated under elevated temperatures and contribute to thermotolerance by enhancing chaperone activity and protein folding efficiency [9,15,50,51,52]. The transcription factors MBF1C and HSFA2 play crucial roles in regulating the expression of chaperones and other stress-responsive enzymes, either directly or indirectly, to facilitate the acquisition of thermotolerance. Moreover, both MBF1C and HSFA2 are regulated by HsfA1s, which are master regulators of the heat stress response and essential components of the complex regulatory network that enables plants to adapt to high-temperature environments [53].

Heat stress tolerance is a pivotal characteristic for wheat adaptation to changing environmental conditions. One of the most effective strategies to improve thermotolerance in wheat is the incorporation of heat-resistant genes or elite haplotypes into cultivated varieties. In this study, it was demonstrated that *TaCOBL6A2* confers heat tolerance. Among the 175 wheat varieties (lines) analyzed, three haplotypes of *TaCOBL6A2* were identified. Hap1 was the most prevalent, accounting for over half of the varieties (lines) (66.29%). In contrast, the frequencies of Hap2 and Hap3 were markedly lower (25.14% and 8.57% respectively). Wheat seedlings carrying the elite haplotype, Hap1, exhibited superior thermotolerance compared with those with Hap2 or Hap3 (Figure 7), which shared the phenotype of being more sensitive to heat stress. These findings suggest that *TaCOBL6A2* has been subject to positive selection during modern wheat breeding, likely due to its contribution to heat stress adaptation. To facilitate the utilization of Hap1 in breeding programs, we developed two KASP markers (Figure S3B,C, Table S6) for efficient haplotype discrimination. These markers enable the precise incorporation of the favorable Hap1 allele into elite wheat cultivars through marker-assisted selection. This approach provides a valuable tool for developing heat-tolerant wheat varieties, addressing the growing need for climate-resilient crops.

In this study, we identified the heat tolerance function of a novel COBRA-like gene, *TaCOBL6A2*, in wheat. Subcellular localization revealed its presence in the plasma membrane, cell wall, and nucleus, suggesting multifunctional roles. *TaCOBL6A2* expression was strongly induced by heat stress, and its overexpression in *Arabidopsis* significantly enhanced thermotolerance. Transcriptomic analysis linked this improvement to heat response pathways and ROS homeostasis, with the up-regulation of heat shock proteins and antioxidant enzymes. Haplotype analysis identified three variants, with the elite haplotype Hap1 (66.29% frequency), showing superior heat tolerance and elevated antioxidant activity. The KASP markers developed for Hap1 enable the efficient breeding of heat-resilient wheat. These findings establish *TaCOBL6A2* as a critical genetic target for climate-smart crop improvement. In future studies, its molecular networks should be investigated in depth and its efficacy in wheat transgenics validated.

## 4. Materials and Methods

### 4.1. Plant Materials, Growth Conditions and Heat Stress Treatment

The seeds of the wheat cultivar ‘Chuanmai 42’were cultivated in a growth chamber under controlled conditions, i.e., 22/18 °C (day/night), 16 h/8 h (light/dark), and 60% relative humidity, for 7 days, up to the one-leaf stage. The seedlings were transferred to growth chambers set to 4 °C (cold stress) or 42 °C (heat stress) for temperature stress treatments, while drought and salt treatments were performed by transferring the seedlings to Petri dishes containing 30% (*w*/*v*) polyethylene glycol (PEG)-6000 or 250 mM NaCl, respectively. Control seedlings were maintained under the original growth conditions. Each treatment included three biological replicates, with each replicate consisting of at least six seedlings.

For heat tolerance evaluation in wheat, nine common wheat varieties/lines, i.e., 21Pin1-1, 21Pin2-1, Chuanmai42, 21PL4939, 21PL5110, Chuanmai 83, H2017, 203137, and 21230, were grown in a growth chamber under the conditions of 20/18 °C (day/night), 14 h/12 h (light/dark), and 60% relative humidity until the two-leaf stage (14 days). The seedlings were then subjected to continuous heat stress at 40 °C for 7 days under normal light and humidity conditions, while the control seedlings continued to grow under normal conditions. Each variety/line was evaluated with three biological replicates, each containing at least four seedlings.

The heat stress treatment of *Arabidopsis thaliana* was performed as described by Silva-Correia et al. [54]. Seeds from the Col-0 ecotype and three overexpression (OE) lines were surface-sterilized and then treated in a 50 °C water bath for 60 min before germination. The seeds were sown on agar plates containing half-strength Murashige and Skoog (MS) medium supplemented with 1% sucrose and 0.8% agar (pH 5.8). After stratification at 4 °C for at least two days, the seedlings were grown in a growth chamber with a 14 h photoperiod and a 22 °C/18 °C (day/night) temperature cycle. Radicles and green cotyledons emergence rates were recorded daily until day 14. Control seeds were germinated under the same conditions without the heat treatment. The germination rate was calculated as the percentage of radicle emergence seeds relative to the total number of seeds.

### 4.2. Arabidopsis Thaliana Transformation

The coding region of *TaCOBL6A2* was amplified with PCR and fused into the binary vector pBI121-GUS by using *Xba*I and *Sac*I restriction sites downstream of the CaMV 35S promoter. The recombinant plasmid, p35S::TaCOBL6A2, was constructed by using In-Fusion Snap Assembly Master Mix (TaKaRa Biotechnology, Dalian, China). The resulting construct was used in the transformation of *A. thaliana* ecotype Col-0 with the floral dip method [55]. Positive transformants were selected on kanamycin-containing plates and confirmed with the PCR amplification of the *TaCOBL6A2* coding sequence. Homozygous lines were obtained at the T_3_ generation by screening at least 100 seeds per line on the kanamycin-containing plates, followed by growth in a controlled-environment chamber.

### 4.3. RNA-Seq Analysis

Twelve-day-old seedlings of *Arabidopsis* Col-0 and three *TaCOBL6A2* overexpression lines, subjected to seed heat treatment, were collected for RNA-seq analysis. Untreated seedlings served as controls. Three biological replicates were prepared for each group, and RNA sequencing was performed by Novogene Bioinformatics Technology Co., Ltd. (Beijing, China). Differential expression analysis, Gene Ontology (GO) enrichment, and KEGG pathway analysis were conducted as described by Deng et al. [56]. Briefly, the differential expression analysis was performed by using the DESeq2R package (v1.20.0). The resulting *p*-values were adjusted by using the Benjamini–Hochberg approach [57] for controlling the false discovery rate (FDR). Genes with an adjusted *p*-value ≤ 0.05 found with DESeq2 were defined as differentially expressed genes (DEGs). The GO enrichment analysis of the DEGs was performed by using the clusterProfiler R package, with gene length bias correction. GO terms with corrected *p*-values < 0.05 were considered significantly enriched. The KEGG pathways enrichment analysis was also conducted by using the clusterProfiler R package to identify significantly enriched pathways.

### 4.4. Protein Feature and Phylogenetic Analyses

Signal peptide predictions were performed with SignalP 5.0 (https://services.healthtech.dtu.dk/service.php?SignalP-5.0 (accessed on 8 October 2022)) [58]. NetGPI-1.1 (https://services.healthtech.dtu.dk/service.php?NetGPI-1.1 (accessed on 8 October 2022)) was used for the prediction of the GPI modification site (ω-site). The predictions of secreted protein signals, subcellular localization, and N-glycosylation sites were performed with TargetP1.1 (http://www.cbs.dtu.dk/services/TargetP/ (accessed on 22 September 2022)), PSORT (https://psort.hgc.jp/ (accessed on 22 September 2022)), and NetNGlyc 1.0 (https://services.healthtech.dtu.dk/service.php?NetNGlyc-1.0 (accessed on 22 September 2022)), respectively. Cis-acting regulatory elements (CAREs) were predicted in the 2-kb upstream region of *TaCOBL6A2* and its homologous genes by using the Plant CARE online server (http://bioinformatics.psb.ugent.be/webtools/plantcare/html/ (accessed on 15 April 2025)).

The COBL protein sequences of *A. thaliana*, *O. sativa,* and *Z. mays* were obtained from the literature [27,34,59], and their protein loci are listed in Table S7. The phylogenetic tree of all the COBLs was constructed with MEGA7.0.2 [60] by using neighbor-joining (NJ) algorithms with the following parameters: Jones–Taylor–Thornton (JTT) model, pairwise deletion, and bootstrap with 1000 replicates.

### 4.5. Subcellular Localization

The coding sequence (CDS) of *TaCOBL6A2*, excluding the stop codon, was inserted into the pBI121-GFP vector by using the *Xba*I and *Bam*HI restriction sites. The resulting TaCOBL6A2–GFP fusion construct was co-transformed into five-week-old tobacco leaves with either the plasma membrane marker OsPIP1;3-mCherry or the nuclear marker OsMADS57-mCherry. After 36 h of dark incubation, the fluorescence signals were visualized by using a laser confocal scanning microscope (TCS SP8, Leica, Wetzlar, Hesse, Germany). The tobacco leaves were treated with 1 M mannitol for 10 min prior to imaging to induce plasmolysis. The co-transformations of the pBI121-GFP and OsPIP1;3-mCherry vectors and of the pBI121-GFP and OsMADS57-mCherry vectors were used as positive controls.

### 4.6. Haplotype Analysis

Natural variation in *TaCOBL6A2* was analyzed by using sequencing data from 278 wheat accessions [61]. Variants located in the coding regions, introns, and untranslated regions (UTRs) of the transcript were selected for haplotype analysis. For intronic variants, only those predicted to potentially affect alternative splicing with snpEff (version 4.3) [62] were retained, while functionally neutral variants were filtered out. Two KASP markers, DQK015 and DQK016, were designed based on variant 1 and variant 2, respectively, by using the WheatOmics 1.0 online tool [63] for haplotype identification.

A total of 175 varieties (lines) were used for dominant haplotype detection and heat tolerance evaluation. Genomic DNA was extracted from seedling leaves by using the SDS method [64], and DNA concentration and quality were assessed by using a NanoDrop 2000^TM^ Spectrophotometer (Thermo Fisher Scientific, Waltham, MA, USA). PCR amplification was performed in a 10 μL reaction volume containing 100 ng of DNA, 1.4 μL of mixed primers, and 5 μL of HiGeno 2 × ProbeMix (Beijing Jiacheng Biotechnology, Beijing, China), following the manufacturer’s protocol. The amplification products were subjected to fluorescence detection at 35 °C for 30 s by using ABI QuantStudio^TM^ 6 Flex (Applied Biosystems, Foster City, CA, USA) to obtain the genotyping results.

### 4.7. RNA Isolation and Real-Time PCR Analysis

Total RNA was extracted from wheat and *Arabidopsis* seedlings by using Trizol reagent (Invitrogen, Carlsbad, CA, USA) following the manufacturer’s protocol. RNA concentration and purity were measured by using a NanoDrop^TM^ 2000 spectrophotometer (Thermo Fisher Scientific, Waltham, MA, USA). First-strand cDNA was synthesized from 1 µg of total RNA by using the RevertAid^TM^ Master Mix (Thermo Fisher Scientific) according to the manufacturer’s instructions. Quantitative RT-PCR (qRT-PCR) was performed on the QuantStudio^TM^ 6 Flex Real-time PCR system (Applied Biosystems, CA, USA) in a 20 µL reaction volume containing 10 µL of SYBR^TM^ Green Master Mix (Applied Biosystems, Foster City, CA, USA) and 5 pmol of each primer. Each sample was analyzed in three technical replicates. The amplification of the wheat *α-tubulin* gene was used as an internal control to normalize the data. The relative expression was estimated by employing the 2^−∆∆CT^ method [65]. Gene-specific primers were designed using DNAMAN v9.0 software, and their sequences are listed in Table S6.

### 4.8. Determination of Antioxidant Enzyme Activity

Approximately 0.5 g of fresh leaf samples was precisely weighed and ground into a fine powder by using liquid nitrogen. The powder was homogenized in 5 mL of pre-chilled extraction buffer (50 mM HEPES-NaOH, pH 7.4, containing 20% (*v*/*v*) glycerol, 1 mM EDTA, 1 mM ascorbic acid (AsA), 1 mM glutathione (GSH), 5 mM MgCl₂, and 1 mM dithiothreitol (DTT)). The homogenate was centrifuged at 10,000× *g* for 30 min at 4 °C, and the supernatant was collected as the enzyme extract for subsequent assays. Antioxidant enzyme activities were measured as follows: superoxide dismutase (SOD) activity was determined by using the nitroblue tetrazolium (NBT) photoreduction assay [66], catalase (CAT) activity with the ultraviolet absorption method [67], ascorbate peroxidase (APX) activity with the ascorbate redox protocol [68], and peroxidase (POD) activity with the guaiacol-based assay method [69].

## Data Availability

The original contributions presented in this study are included in the article/Supplementary Materials. Further inquiries can be directed to the corresponding author(s).

## Supplementary Materials

The following supporting information can be downloaded at: https://www.mdpi.com/article/10.3390/ijms26094101/s1.
