# Supplementary material for: Wheat COBRA-like Gene TaCOBL6A2 Confers Heat Tolerance in Plants
Source: Int J Mol Sci. 2025 Apr 25;26(9):4101. doi: 10.3390/ijms26094101 (PMC12071834; doi:10.3390/ijms26094101)
Supplement: Supplementary file 1 [file ijms-26-04101-s001.zip › Supplementary Materials/Supplementary Figure S1-S4.pdf]

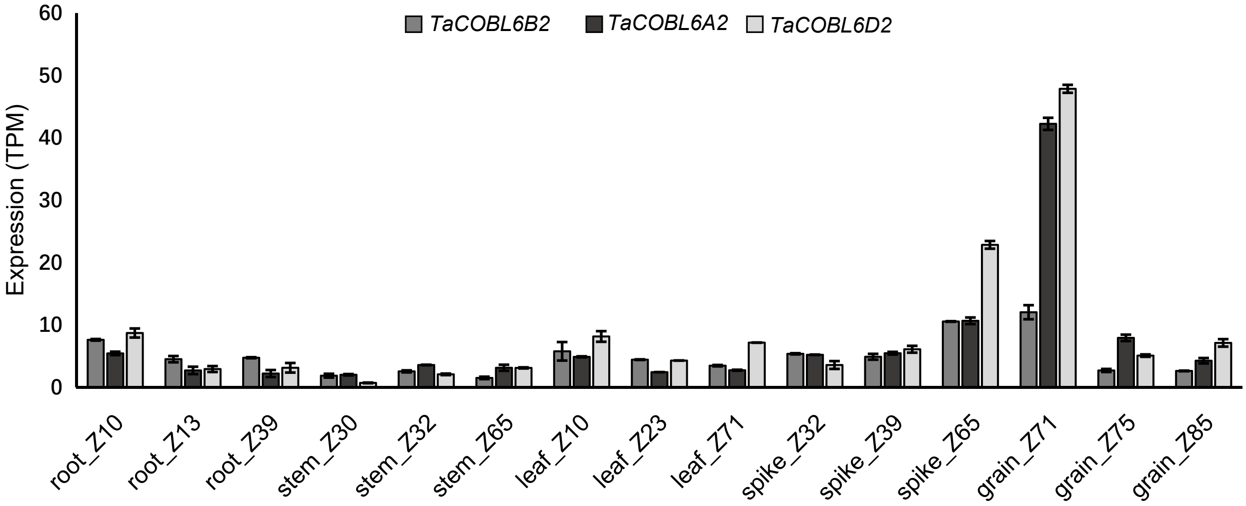

Figure S1. Expression profiling of *TaCOBL6A2* and its homologous genes in wheat.

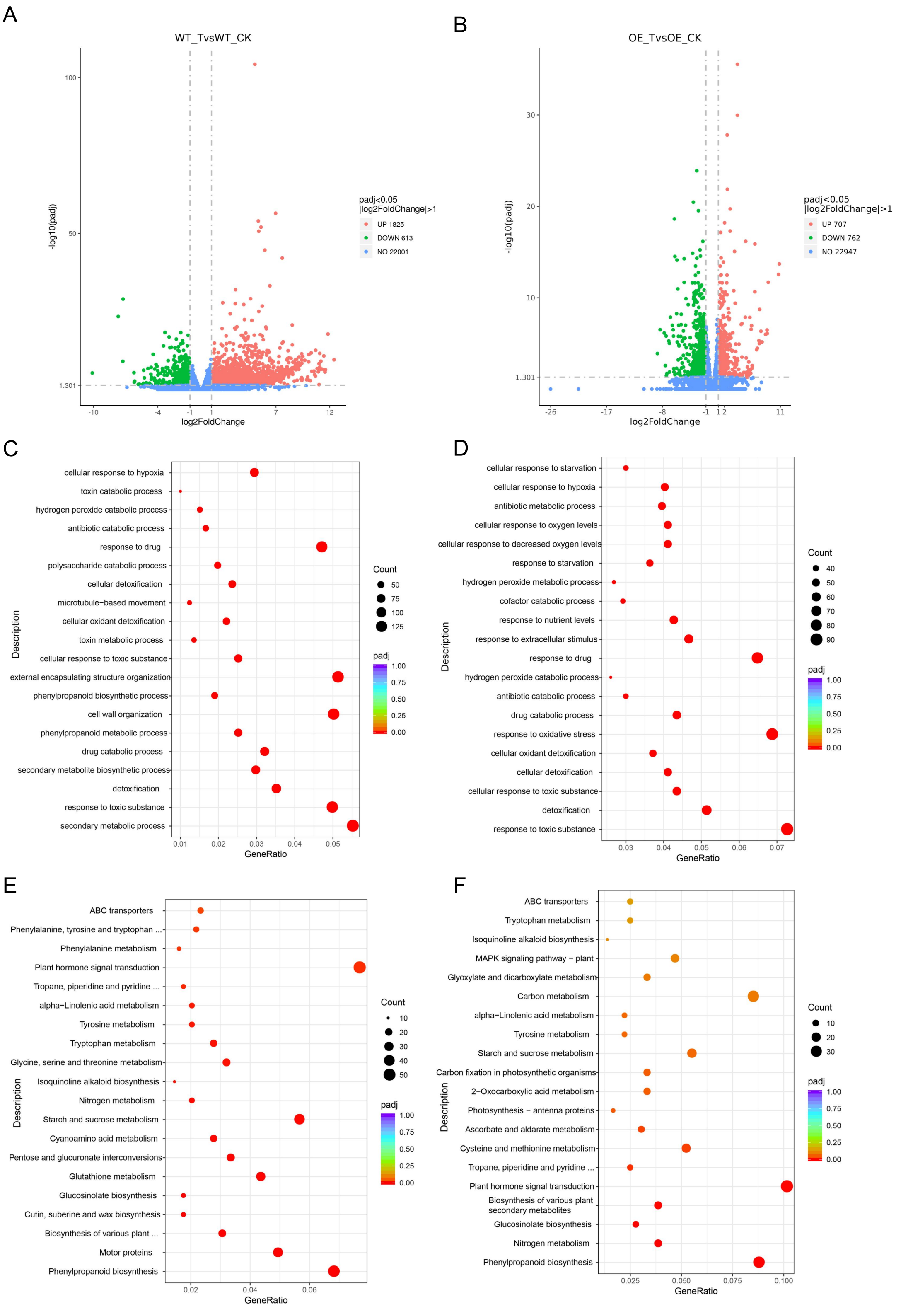

Figure S2. Functional enrichment analysis of differentially expressed genes (DEGs) under heat stress. (A-B) Volcano plots of DEGs in wild-type (A) and *TaCOBL6A2* overexpression lines (B). Significantly up-regulated genes (red), down-regulated genes (green), and non-significant genes (blue) are highlighted. (C-D) Top 20 significantly enriched Gene Ontology (GO) terms in wild-type (C) and overexpression lines (D) under heat stress. (E-F) Top 20 enriched KEGG pathways in wild-type (E) and overexpression lines (F) under heat stress.

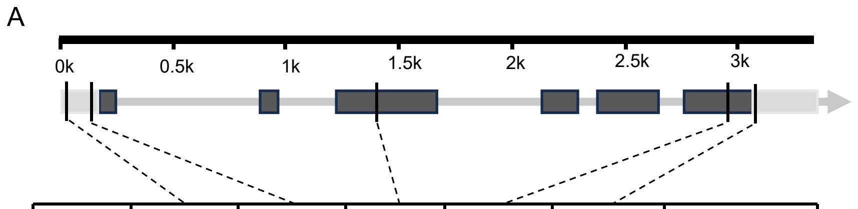

Hap	Var 1	Var 2	Var 3	Var 4	Var 5	varieties (lines)
Hap 1	T	T	A	A	T	116
Hap 2	C	C	G	G	C	44
Hap 3	C	T	G	G	T	15

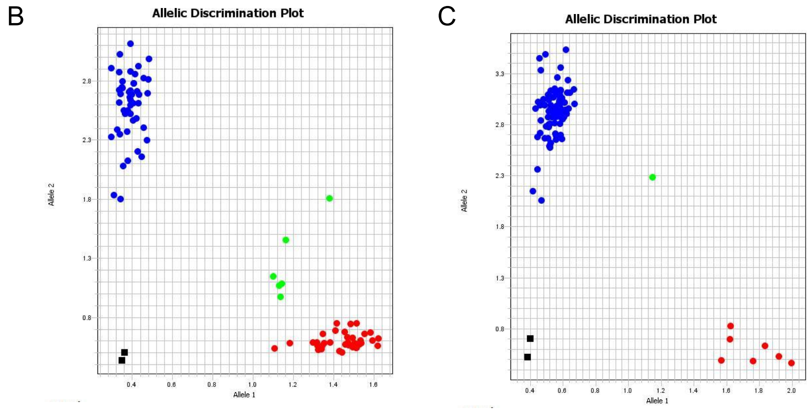

Figure S3. Gene structure and haplotype analysis of *TaCOBL6A2* in wheat. (A) Haplotype identification across 175 wheat varieties (lines). Grey boxes, black boxes, and grey lines represent UTR regions, exons, and introns, respectively. (B-C) Genotyping of wheat varieties (lines) using KASP markers DQK015 (B) and DQK016 (C). Blue dots represent homozygous allele A/A, red dots represent homozygous allele G/G, and green dots represent heterozygous allele A/G. Black squares indicate no-template controls.

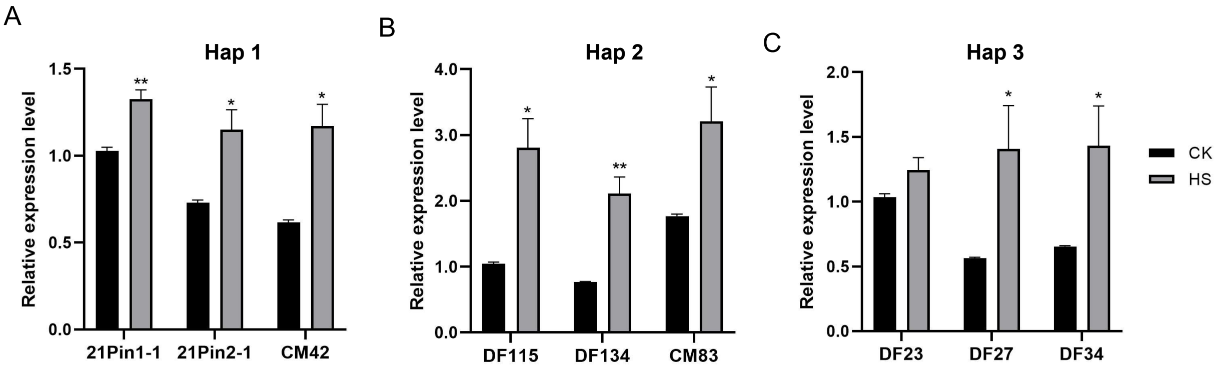

Figure S4. The expression pattern of *TaCOBL6A2* in different haplotype varieties (lines) under heat stress treatment. (A-C) Relative expression levels of *TaCOBL6A2* in Haplotype 1 (Hap 1), Haplotype 2 (Hap 2), and Haplotype 3 (Hap 3) varieties (lines), respectively. Asterisks indicate significant differences between haplotypes (*$p < 0.05$; **$p < 0.01$).
